# Titanium surface modification by using microwave-induced argon plasma in various conditions to enhance osteoblast biocompatibility

**DOI:** 10.1186/s40824-015-0034-2

**Published:** 2015-05-10

**Authors:** Gyeung Mi Seon, Hyok Jin Seo, Soon Young Kwon, Mi Hee Lee, Byeong-Ju Kwon, Min Sung Kim, Min-Ah Koo, Bong Joo Park, Jong-Chul Park

**Affiliations:** Cellbiocontrol Laboratory, Department of Medical Engineering, Yonsei University College of Medicine, 134 Shinchon-dong, Seodaemun-gu, Seoul, 120-752 Republic of Korea; Brain Korea 21 PLUS Project for Medical Science, Yonsei University College of Medicine, 134 Shinchon-dong, Seodaemun-gu, Seoul, 120-752 Republic of Korea; Department of Electrical and Biological Physics and Plasma Bioscience Research Center, Kwangwoon University 305 Dasanjae, 20 Kwangwoongil, Nowon-gu, Seoul, 139-701 Republic of Korea

**Keywords:** Titanium, Microwave-induced argon plasma, Surface modification, Biocompatibility

## Abstract

**Background:**

Titanium is a well proven implantable material especially for osseointegratable implants by its biocompatibility and anti-corrosive surface properties. Surface characteristics of the implant play an important role for the evolution of bone tissue of the recipient site. Among the various surface modification methods, plasma treatment is one of the promising methods for enhance biocompatibility. We made microwave-induced argon plasma at atmospheric pressure to improve in titanium surface biocompatibility.

**Results:**

Various states of emission spectra from excited species-argon, nitrogen atoms and oxygen atoms were observed. The electron energy band structures are the unique characteristics of atoms and functional groups. Microwave-induced argon plasma treatment changed the titanium surface to be very hydrophilic especially on the 5 s short treatment and 30 s, 90 s long treatment samples that detected by contact angle measurement. MC3T3-E1 attachment and proliferation assay significantly increased in 5 s at short treatment, 30 s, and 90 s at long treatment after 5 days incubation.

**Conclusions:**

Result indicated that microwave-induce argon plasma treatment would be an effective method to modify titanium surface for enhancing cell-material interactions.

## Background

The success of a dental implant is based on the osseointegration that is defined as the direct contact between the bone tissue and the dental implant surface, without fibrous tissue growing at the interface [1,2]. Titanium is a well proven implantable material especially for dental and orthopedic implants [3–5]. The popularity of titanium for osseointegratable implants is based on its biocompatibility and anti-corrosive surface properties. Surface characteristics of the implant play an important role for the evolution of bone tissue of the recipient site, after implantation [6,7]. In order to further enhance its biocompatibility, several special treatments to create surfaces such as sandblasted large-grit and acid-etched (SLA) surface, resorbable blasted media (RBM) surface and micro-arc oxidized (MAO) surfaces were conventionally used.

Among the various surface modification methods, plasma treatment is one of the promising methods. Diverse functional groups could be introduced to the surface by various appropriate plasma sources. These functional groups improve biocompatibility of the surface and enhance cellular responses [8]. An atmospheric plasma system is commonly used for sterilization and surface modification. As previously, our laboratory made microwave-induced argon plasma at atmospheric pressure. This system provided good sterilization effects on microorganisms, fungi, Escherichia coli, Methicillin-resistant staphylococcus aureus and mycotoxins [9–12]. Also, surface modification effects on various biomaterials such as PLGA, polyurethane and silk fibroin were investigated by microwave-induced argon plasma [13–15].

In this study, microwave-induced argon plasma was adopted to enhance titanium surface biocompatibility, and different distance from nozzle and treating time were controlled to confirm the effects of this plasma system. Distances between sample and nozzle were set to 3 cm and 7 cm. For convenience, ‘short treatment’ stands for the test of 3 cm distance and ‘long treatment’ stands for the test of 7 cm distance.

## Methods

### Materials

Thickness and diameter of the CP-2 titanium (Ti) specimens (Seoul Titanium Inc., Korea) were 0.3 mm and 13 mm. The specimens were solvent-cleaned by a serial ultrasonic treatment in acetone, methyl alcohol and de-ionized water, and then dried with oxygen blow.

### Treatment of microwave-induced argon plasma

Self-designed microwave-induced argon plasma was used in this study as previously described [9]. Briefly, this system consisted of 2.45 GHz magnetron power supply (1 kW), an applicator including a tuning section and the nozzle made of quartz. The microwave was introduced through a WR-284 copper waveguide with internal cross section dimensions of 72 mm × 24 mm. The plasma generated at the end of a nozzle was formed by an interaction between the high electrical fields, which is generated by the microwave power supply, the waveguide aperture and the gas nozzle. The electric field intensity around the nozzle was calculated by a high frequency structure simulator (HFSS) code simulation as previously described [11]. Argon was used as a working gas for this plasma system, which was chosen for its inertness, and the gas flow rate is approximately 100 min^−1^ at 8 kg_f_/cm^2^. For evaluating the plasma efficiency on Ti disks, Ti disks were placed in front of a nozzle in the plasma system and exposed to plasma for 1 s, 5 s at ‘short treatment (3 cm)’ and 10 s, 30 s, 90 s at ‘long treatment (7 cm)’, respectively.

### Measurement of emission spectra

The emission spectra from the plasma according to the gas type were acquired through the optical fiber in the range of 200-1200 nm wavelength. The optical emission spectrometer (HR4000, Ocean Optics Inc, USA) was used to analyze the emission spectra and the electronically excited species generated by the plasma device. The measurement point has been set to be just region of plasma plume. The optical emission spectrum was obtained from the plasma device.

### Contact angle measurement

Contact angle was measured using the sessile drop method [16]. to evaluate hydrophilicity of prepared specimens. De-ionized water was utilized as probing liquid. Surface energy was also measured by same method. Three samples were used to collect the contact angle data in diiodomethane, formamide and glycerol for each plasma treated titanium. Lewis acids and bases were used to calculate the surface energy [17].

### Cell culture

MC3T3-E1 mouse pre-osteoblastic cell line (Riken, Japan) was maintained in αMEM medium modified with ascorbic acid supplemented with 10 % fetal bovine serum and 1 % antibiotic antimycotic solution (WelGENE, Korea). Cells were cultured at 37 °C in a humidified 5 % CO_2_ atmosphere incubator.

### Cell attachment and proliferation test

Microwave-induced argon plasma treated Ti disks were placed to 24 well plate and the titanium disks were sterilized in 70 % ethanol for 30 min and washed three times in distilled water. After sterilization, MC3T3-E1 cells were seeded on titanium disks at a density of 6 × 10^4^ cells/disk and incubated for 2 h for cell attachment test. For cell proliferation test, cells were seeded at a density of 3 × 10^4^ cells/disk and cultured for 1, 3, 5 days in a CO_2_ incubator. The viability of the cells was determined with 3-(4,5-dimethylthiazol-2-yl)-2,5-diphenyltetrazolium bromide (MTT) assay. Briefly, MTT was added to each well at 500 μg/mL and further incubated for 4 h at 37 °C. After incubation, dimethyl sulfoxide and glycine buffer was added to each well for the solubilization of the formed formazan salts. The absorbance of each well was measured spectrophotometrically at 570 nm by a microplate reader.

### Cell morphometric analysis

For cell morphometric analysis, the cells were seeded at the initial seeding density of 1.0 × 10^4^ cells/disk and after 2 h of incubation they were fixed with ice cold 70 % ethanol. Cell plasma membrane was visualized by staining with Texas Red C2-maleimide (Texas Red, 30 ng/ml in PBS, Invitrogen) [18]. Cell nuclei were counterstained with Hoechst 33258 (1 g/ml in PBS, Sigma). Ten random pictures were taken by IX-70 microscope, equipped with a DP-71 digital camera (Olympus, Japan). Cell attachment area, perimeter and Feret’s diameter of 30 cells for each group were measured by Image J software (NIH, USA) [19].

### Statistical analysis

All results were statistically analyzed by Student *t*-test. Data are expressed as mean ± standard error of mean. P values <0.05 were considered statistically significant.

## Results

### Optical emission spectroscopy (OES)

**Fig. 1:**
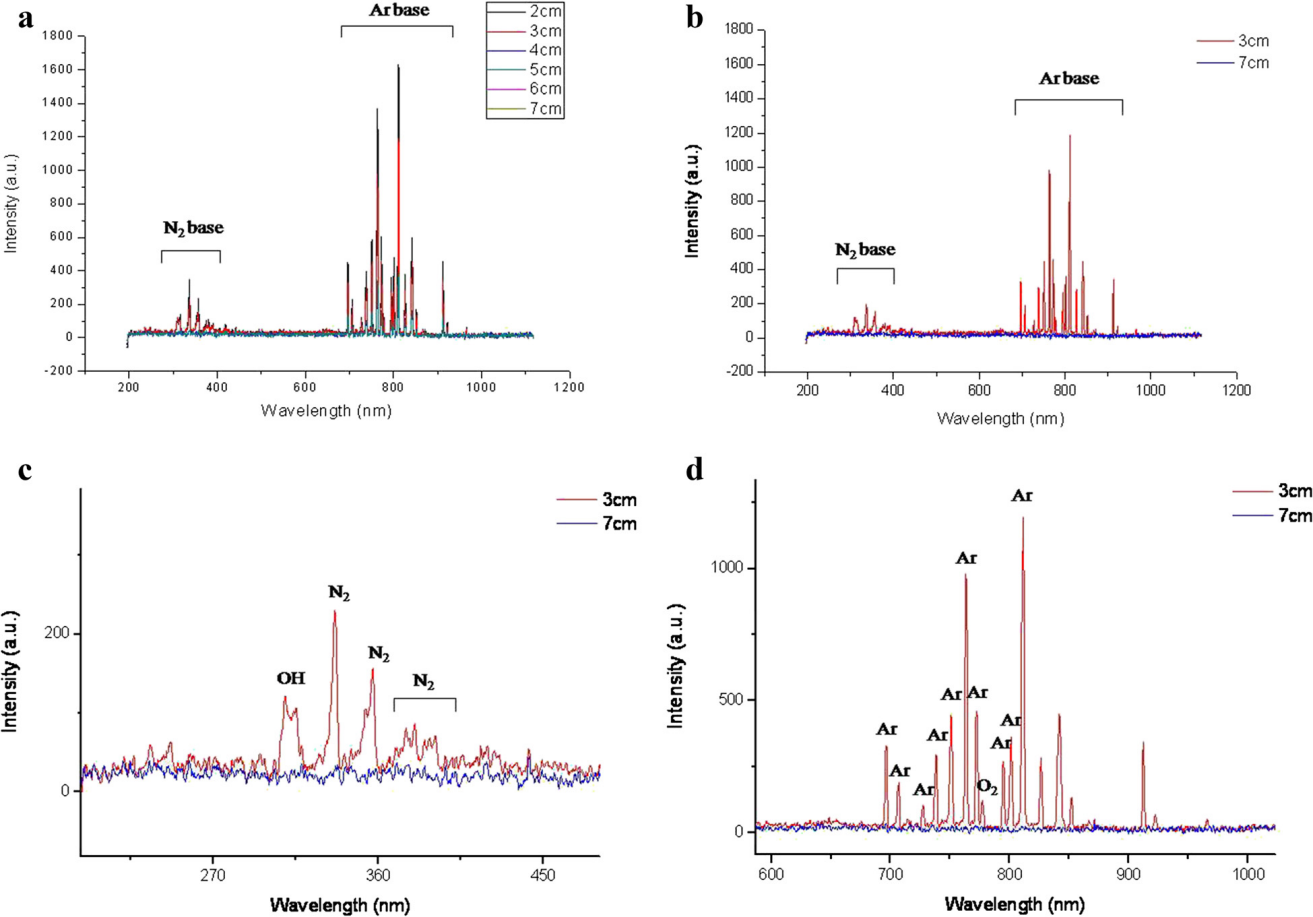
Emission spectra of microwave-induced argon plasma. (**a**) Comparison of plasma spectra exited in different distance from nozzle. (**b**) Different emission spectra between ‘short treatment’ and ‘long treatment’. (**c**) Emission spectra of N2 base. (**d**) Emission spectra of Ar base

The emission spectra of the microwave-induced argon plasma were collected by optical emission spectroscopy (OES) in order to probe the existence of excited nitrogen derivatives. Various states of emission spectra from excited species-argon atoms (696, 706, 727, 738, 750, 763, 772, 795, 801, 811 nm), nitrogen atoms (337, 357, 373, 378 nm) and oxygen atoms (777 nm) were observed and the spectra were depicted in Fig. 1. Excited OH molecular emissions around 308 nm were also observed [20–23].

### Contact angle measurement

**Fig. 2:**
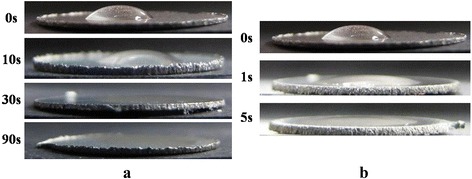
Change of hydrophilicity on plasma treated titanium surface. (**a**) Long treatment group (7 cm). (**b**) Short treatment group (3 cm)

Changes of contact angle and surface energy on the plasma treated titanium surfaces were summarized in Tables 1 and 2. The water contact angle of untreated titanium surface was 54.8° and it fell to nearly zero degree on the 90 s treated sample at ‘long treatment’. Figure 2 shows change of contact angle by de-ionized water drop. When titanium sample was placed in the short treatment, 5 s treated samples showed similar effects to 30 s treatment in the long treatment. The results of surface energy provide more detailed comparison between ‘short treatment’ and ‘long treatment’ (Tables 1 and 2). The surface energy of 5 s treated titanium at ‘short treatment’ was 1026.74 mJ/m^2^ and the results of 30 s and 90 s treatments at ‘long treatment’ were 953.65 mJ/m^2^ and 1274.84 mJ/m^2^, repectively. Put differently, the effect of 5 s treatment at ‘short treatment’ was similar to the result of 30 s or 90 s at ‘long treatment’. Even though OES could not detect any emission spectra at ‘long treatment’, radicals and photons were still existed at ‘long treatment’.

**Table 1:** Long treatment condition; contact angle and surface energy on plasma treated titanium (n = 3)

	0 s	10s	30s	90s
Formamide (°C)	39.06	0	0	0
Glycerol (°C)	44.09	18.69	14.47	8.60
Diiodomethane (°C)	26.70	22.57	19.89	15.91
Surface energy (mJ/m^2^)	791.53	672.07	953.65	1274.84

**Table 2:** Short treatment condition; contact angle and surface energy on plasma treated titanium (n = 3)

	0 s	1 s	5 s
Formamide (°C)	39.06	0	0
Glycerol (°C)	44.09	15.08	13.57
Diiodomethane (°C)	26.70	22.35	18.14
Surface energy (mJ/m^2^)	791.53	895.10	1026.74

### MC3T3-E1 attachment and proliferation

**Fig. 3:**
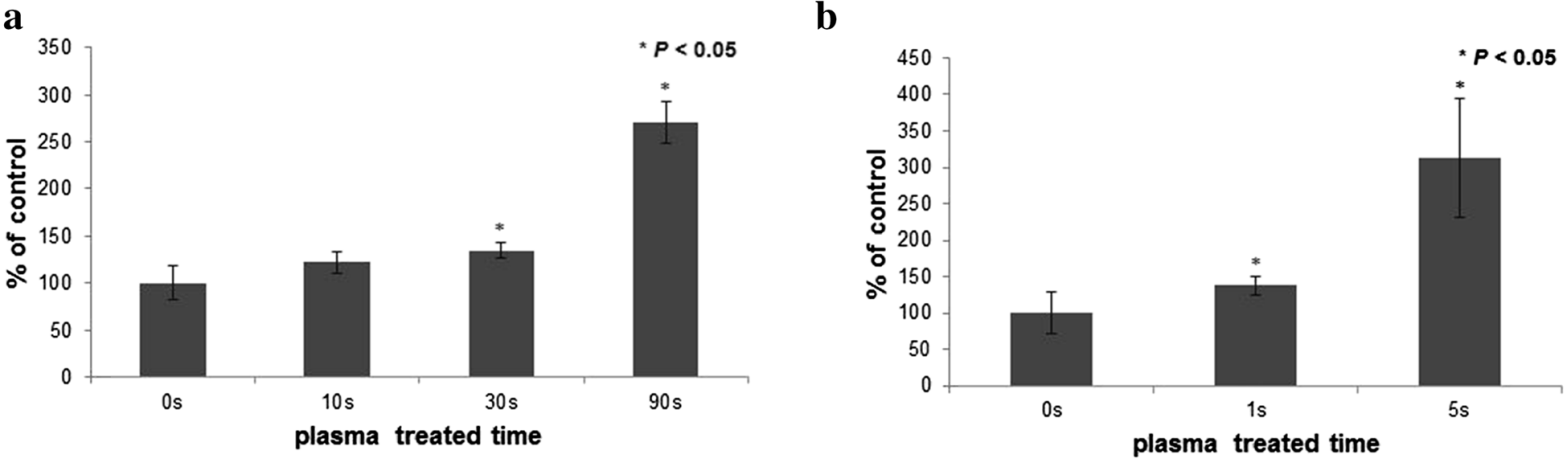
MC3T3-E1 cell attachment on plasma treated titanium surface by plasma treated time. (**a**) Long treatment condition; MC3T3-E1 attachment test on titanium surface for 2 h by MTT assay (**b**) Short treatment condition; MC3T3-E1 attachment test on titanium surface for 2 h by MTT assay

The number of attached MC3T3-E1 cells cultured for 2 h on the plasma treated titanium was showed in Fig. 3. When titanium was placed under the long treatment, 30 s and 90 s treatments are positively effective for attachment. The 30 s and 90 s treated samples showed significantly enhanced attachment compared to control (0 s). When titanium was placed under the short treatment, cell attachment was significantly enhanced after 5 s treatment.

**Fig. 4:**
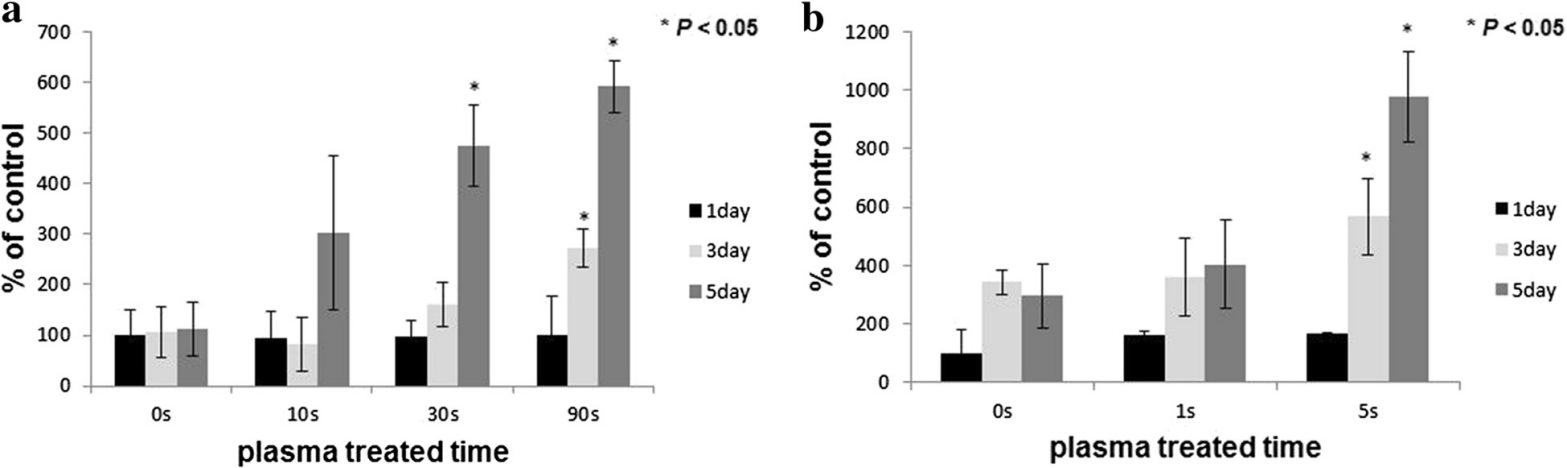
MC3T3-E1 cell proliferation on plasma treated titanium surface by plasma treated time. (**a**) Long treatment condition; MC3T3-E1 proliferation test on titanium surface by MTT assay (**b**) Short treatment condition; MC3T3-E1 proliferation test on titanium surface by MTT assay

Enhanced proliferation patterns of MC3T3-E1 cultured for 1, 3 and 5 days on the plasma treated titanium were plotted in Fig. 4. After 3 days incubation, the number of cells at ‘long treatment’ was significantly increased after 90 s plasma treatment and the number of cells after 30 s treatment was also significantly increased after 5 days incubation. Moreover, in the short treatment test, cell proliferation was increased just in 5 s treatment time.

### Cell morphometric analysis

**Fig. 5:**
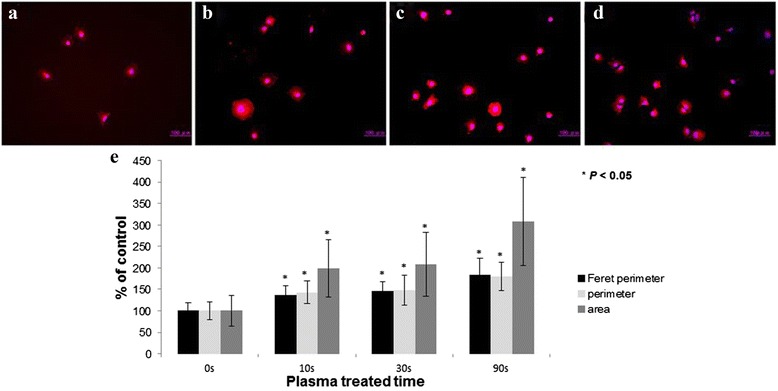
Long treatment condition; Change of morphology of attached MC3T3-E1 cells on plasma treated titanium. (**a**) Untreated sample. (**b**) 10 s treated sample. (**c**) 30 s treated sample. (**d**) 90 s treated sample. (**e**) Relative morphometric analysis of attached MC3T3-E1 cells on plasma treated titanium

**Fig. 6:**
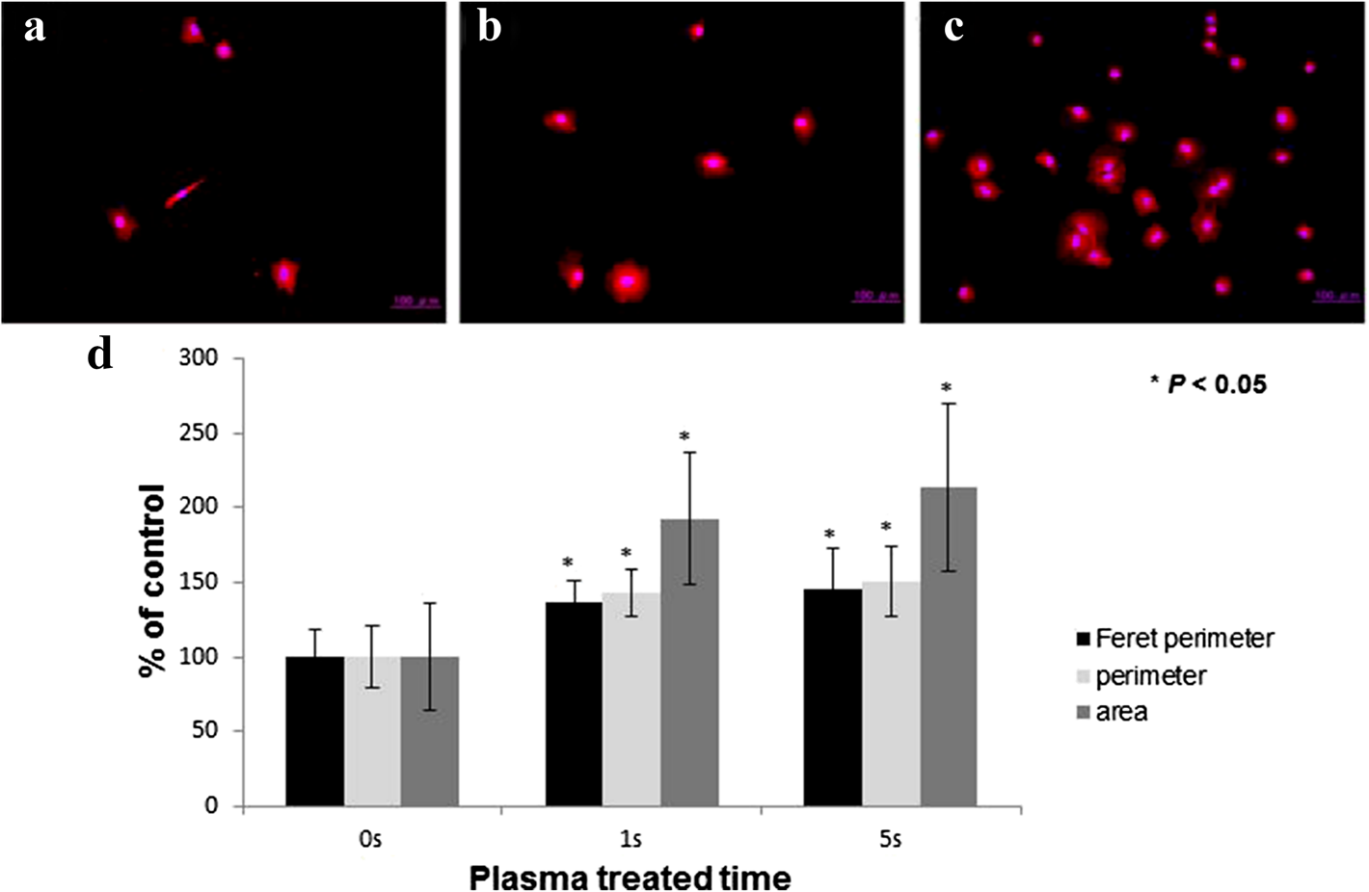
Short treatment condition; Change of morphology of attached MC3T3-E1 cells on plasma treated titanium. (**a**) Untreated sample. (**b**) 1 s treated sample. (**c**) 5 s treated sample. (**d**) Relative morphometric analysis of attached MC3T3-E1 cells on plasma treated titanium

Fluorescence images of cells on the titanium disks visualizing the plasma membrane by Texas Red maleimide staining have shown that, the attached cells on plasma treated samples were increased in population and also obviously enlarged. Cells adhering to non-treated disks were smaller and round-shaped, while cells on microwave induced plasma treated disks occupied larger attachment area, well-spread elongated morphology with formed lamellipodia-like projections (Figs. 5 and 6). Population of cells also increased after plasma treatment. This result means that the ratio of initially attached cells increased after plasma treatment.

The area, perimeter and Feret’s diameter of the attached MC3T3-E1 were measured and plotted in Figs. 5 and 6. All of the measured parameters increased significantly on the plasma treated titanium regardless of the treating time and treating condition.

## Discussion

Gaseous plasma provides a complex body of radicals, ions, electrons, and photons which are in various excited energy states. The optical emission spectra from the energy quenching processes of the excited species can be performed to characterize the plasma. The electron energy band structures are the unique characteristics of atoms and functional groups. Since the atmospheric pressure discharge was fired at ambient atmosphere, the emissions from not only excited argon atoms but also excited nitrogen and oxygen atoms and OH molecules could be detected. The emission spectra of the microwave-induced argon plasma were collected by optical emission spectroscopy (OES) in order to probe the existence of excited nitrogen derivatives. Compared to the short treatment, the long treatment could not be detected by OES. OES can observe the optical atmosphere and the long treatment cannot provide optical condition. Although emission spectra of the long treatment were not detected, radicals and electrons were still existed at the atmosphere. This phenomenon is easily understood by contact angle analysis.

Microwave-induced argon plasma treatment changed the titanium surface to be very hydrophilic. In general, surface hydrophilicity is very important for homogeneous and sufficient surface-compatibility as well as for good growth of cells on polymer materials [24]. Furthermore, it was reported that enhanced fibronectin adsorption and reduced albumin adsorption occurs on hydrophilic surfaces and these elongated fibronectin conformation on the hydrophilic surface was effected to cell attachment [19]. This plasma system could produce super-hydrophilic surface even for the short period time in the short treatment condition. In the long treatment condition, the surface had changed to super-hydrophilic after 90 s even though the OES was not detected at this point. In case of UV modification, 48 h treatment time is required to decrease the water contact angle of machined surface to less than 10 °. It might be resulted from the high population of various exited species that only short treatment time of microwave-induced argon plasma was required to achieve super-hydrophilic surface, which is a typical characteristic of atmospheric pressure gaseous discharge.

To confirm the effects of this plasma system for cellular responses, different treating conditions were adopted on this study. The initial interaction of cells with the biomaterial plays a key role for and early acceptance of the implant [25]. In previous study, we confirmed that titanium surface treated helium atmospheric pressure glow discharge (He-APGD) promoted selectively higher absortion of fibronectin, a protein of critical importance for cell/biomaterial interaction. Therefore, we expected the improved cell response appeared to be dependent on the fibronectin-integrin mediated mechanism of cell attachment [26]. In this sense, cell attachment and proliferation test was performed on the plasma treated titanium surface. Cell morphometric analysis is also performed to confirm changed cell morphology after plasma treatment. These results support that the microwave induced argon plasma system which was used for this study could be very useful to enhance cellular responses on titanium surface for both the short treatment and the long treatment. Even though this plasma has very strong power which can be used for sterilization microorganisms in a very short time, it can be controlled very easily by changing the position of samples.

## Conclusions

Microwave-induced argon plasma system was employed to modify titanium surface in order to achieve enhanced biological responses. Plasma treated titanium surface turned to be very hydrophilic surface and it did not require more than 90 s treatment time. Surface modified titanium by plasma treatment increased surface energy and it allowed enhancing cell attachment and proliferation on the titanium surface. The increase of cell population, cell area, perimeter, and Feret’s diameter of MC3T3-E1 cells were also promoted. Conclusively, microwave-induce argon plasma treatment would be an effective method to modify titanium surface for enhancing cell-material interactions.
